# Use of real-time three-dimensional echocardiography to assess left ventricular systolic synchronization after dual-chamber pacing therapy

**DOI:** 10.3892/etm.2012.700

**Published:** 2012-09-06

**Authors:** LIN LIU, LIANZHONG ZHANG, SHAOBO DUAN

**Affiliations:** Department of Ultrasound, Henan Provincial People’s Hospital, Zhengzhou 450003, P.R. China

**Keywords:** echocardiography, real-time three-dimensional, pacing, asynchrony, left ventricular

## Abstract

This study was designed to evaluate the left ventricular systolic synchronization in patients implanted with dual-chamber DDD mode cardiac pacemakers by real-time three-dimensional echocardiography (RT3DE). Twenty patients implanted with DDD mode cardiac pacemakers for 12 months and 20 healthy subjects underwent RT3DE. This method provided left ventricular end-diastolic volume (LEDV), left ventricular end-systolic volume (LESV), stroke volume (SV), left ventricular ejection fraction (LVEF), the mean value of the time to minimal systolic volume of the 16 left ventricular segments (Tmean), the standard deviation of Tmean (T-SD), the maximal difference of the time to minimal systolic volume of the 16 left ventricular segments (Tmax) and time-volume curves of the 16 left ventricular segments. Results showed that compared with the healthy group, LESV was significantly increased (P<0.05), SV and LVEF were significantly decreased (P<0.05) and T-SD and Tmax were significantly prolonged (P<0.05) in patients implanted with DDD mode cardiac pacemakers. The time to minimal systolic volume of the 16 left ventricular segments time-volume curves differed in patients implanted with DDD mode cardiac pacemakers. Asynchronization of the left ventricular systolic performance in patients implanted with DDD mode cardiac pacemakers was observed. The results showed that RT3DE is a quantitative method used to evaluate left ventricular systolic synchronization.

## Introduction

An artificial cardiac pacemaker, important in treating cardiac bradycardia, is able not only to stimulate heart contraction, but also to provide a method and instrument of arrhythmia interventional diagnosis and treatment. DDD mode cardiac pacemakers have been applied in the clinic since the end of the 1970s. DDD mode cardiac pacemakers are able to pace and monitor the atria and ventricles, whereas the reactive mode of perception restrains or triggers stimulation.

Real-time three-dimensional echocardiography (RT3DE) is a crucial technical breakthrough in the field of ultrasonic imaging, providing investigators with full-volume data, analysis of the size, shape and function of the cardiac chambers, and a feasible and simple new technique for evaluating heart global and segmental function as well as systolic synchronism (1,2). Previous studies (3) reported the function of patients with coronary artery diseases and hypertensive heart disease who were assessed by RT3DE. The aim of the present study was to investigate the value of RT3DE in patients implanted with DDD mode cardiac pacemakers, including an analysis of the left ventricular systolic function and synchronism.

## Materials and methods

### Study population

The study population included 20 healthy subjects (12 men and 8 women; mean age, 62.8±5.5 years) and 20 patients implanted with DDD mode cardiac pacemakers (9 men and 11 women; mean age, 66.6±7.2 years). The 20 healthy subjects were confirmed by history, physical examination, electrocardiography, X-ray and echocardiography. All of the patients presented with sinus node dysfunction prior to implantation with DDD mode cardiac pacemakers and, according to the results of coronary angiography and echocardiography, were diagnosed with coronary artery disease and other heart diseases. The patients implanted with DDD mode cardiac pacemakers were in a stable haemodynamic condition when included in the study. The maximum cardiac output obtained from echocardiography was set to the optimal atrioventricular delay in DDD pacing. The programmable upper frequency was set to maximal heart rate <(220-age) x90%. According to 24 Holter analysis, ventricular pacing accounts for >80% of the effective pacing. The site of the right ventricular lead was placed at the apex in the right ventricle. Informed consent was obtained from all subjects and the study was approved by our local Ethics Committee (Henan Provincial People’s Hospital, Zhengzhou, China).

### RT3DE

Healthy subjects and patients implanted with DDD mode cardiac pacemakers for 12 months underwent left ventricular measurement by RT3DE in the left lateral decubitus position.

RT3DE was performed with iE33 (Philips, Guildford, Surrey, UK) and X3-1 matrix array transducer. Pyramidal volume, which has a relatively narrow sector width, is obtained in real time. To obtain a larger volume, it is necessary to visualize the entire left ventricle, since full-volume acquisition is used. The optimized apical four-chamber view was obtained during the full volume acquisition. Four real-time volumes may be obtained from consecutive cardiac cycles. Apical full-volume imaging of the left ventricle was obtained for all subjects. Full-volume acquisition is performed during breathhold and requires a stable R-R interval to minimize the stitch artifacts.

Quantitative analysis involved regulating the sagittal and coronal sections, located in the middle of the left ventricle, and the transverse section was laid at the mitral valve loop level at the end-diastole and end-systole separately. Subsequently, five endocardium sample points of the four-chamber view mitral valve loop level’s interventricular septum and lateral wall, and two-chamber view mitral valve loop level’s inferior wall, anterior wall and cardiac apex were selected for the analysis. Q-LAB software (Landwind Scinece & Technology Park, Shiyan Town, Bao’an District, Shenzhen, China)was employed to trace out the outline of the triaxial endocardium automatically. The 16 left ventricular segments were defined by the American Heart Association (4). Left ventricle enddiastole volume (LEDV), end-systolic volume (LESV), stroke volume (SV), eject fraction (LVEF), mean value of the time to minimal systolic volume of the 16 left ventricular segments (Tmean), the standard deviation of Tmean (T-SD), the maximal difference of the time to minimal systolic volume of the 16 left ventricular segments (Tmax) in each patient were calculated from full volume imaging (5).

### Data analysis

RT3DE data were stored and analyzed with Q-LAB quantitative analysis software. To minimize the variability of the measurements, all ultrasonic recordings were performed by the same author. In total, 40 subjects were selected for reliability analyses between two independent observers.

### Statistical analysis

Data were presented as the mean ± SD. All the analyses were performed with SPSS 13.0 for Windows. For the comparison of parametric variables, the independent samples t-test was used. P<0.05 was considered to indicate a statistically significant result.

## Results

### Feasibility

RT3DE recordings were adequate for analysis in all subjects.

### Clinical data and left ventricular function

The difference between the two groups for the clinical and left ventricular function parameters of age, HR, LEDV, LESV, SV and LVEF, are listed in Table I.

No significant differences in age, HR and LEDV were found between the two groups. Compared with the healthy group, LESV was significantly increased (25.27±7.71 vs. 42.95±23.79 ml; P<0.05), SV was significantly decreased (47.87±11.81 vs. 38.43±9.60 ml; P<0.05) and LVEF was significantly decreased (65.22±6.31 vs. 48.08±9.79%; P<0.05) in patients implanted with DDD mode cardiac pacemakers.

### Left ventricular systolic synchronism

**Figure 1:**
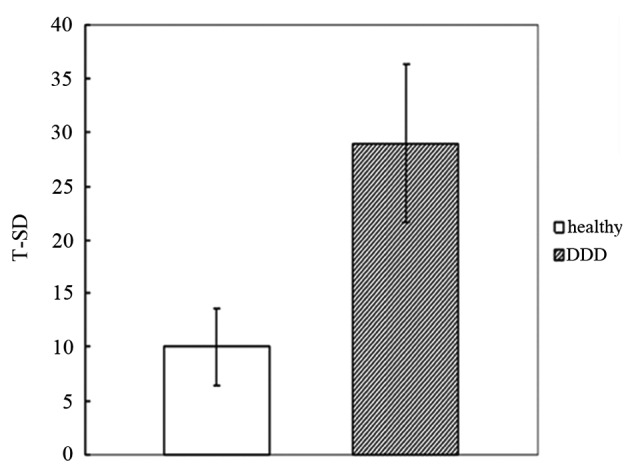
Standard deviation of the mean value of the time to minimal systolic volume of the 16 left ventricular segments (T-SD) between the two groups (P<0.05).

**Figure 2:**
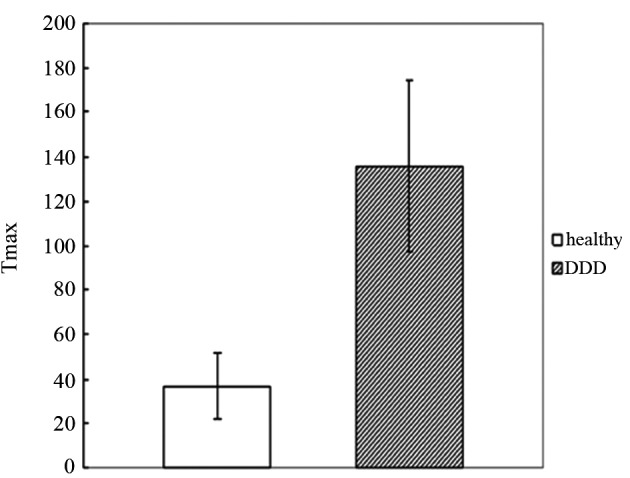
Maximal difference of the time to minimal systolic volume of the 16 left ventricular segments (Tmax) between the two groups (P<0.05).

**Figure 3:**
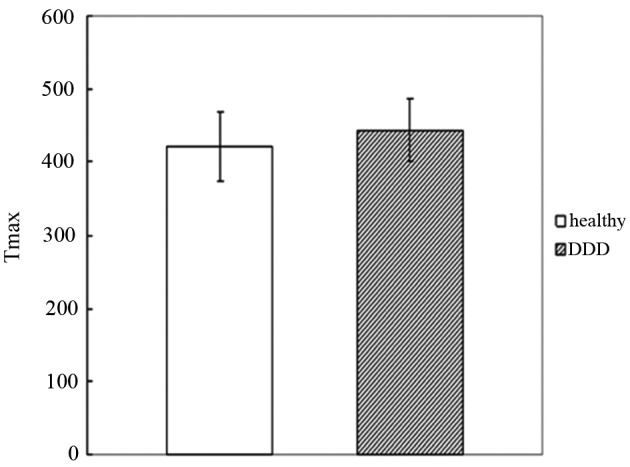
Mean value of the time to minimal systolic volume of the 16 left ventricular segments (Tmean) between the two groups (P<0.05).

Results showed that compared with the healthy group, T-SD (10.00±3.62 vs. 29.00±7.31 msec; P<0.05) (Fig. 1) and Tmax were significantly prolonged (36.60±14.90 vs. 135.70±38.65 msec; P<0.05; Fig. 2) in patients implanted with DDD mode cardiac pacemakers. Significant differences in T-SD and Tmax between the two groups (Table II) were observed, while there were no significant differences in Tmean between the two groups (Fig. 3).

### Time-volume curves of the 16 left ventricular segments

**Figure 4:**
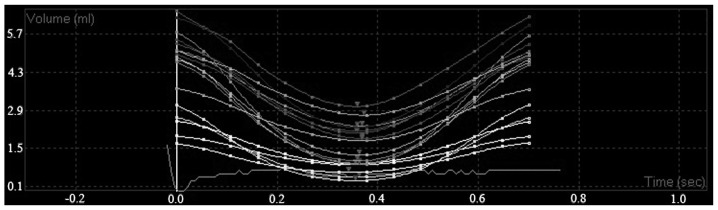
Time-volume curves of the 16 left ventricular segments in healthy subjects appeared ordered and regular, with a homodirectional waveform.

**Figure 5:**
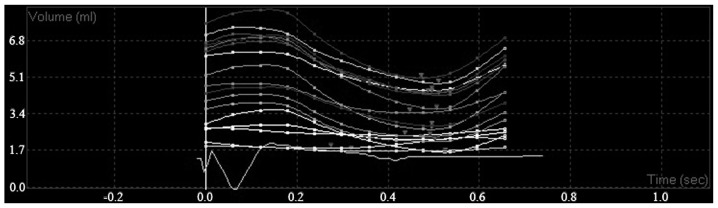
Time-volume curves of the 16 left ventricular segments in patients implanted with dual-chamber mode cardiac pacemakers appeared to be chaotic and irregular, with a heterodirectional waveform.

The time-volume curves of the 16 left ventricular segments appeared ordered and regular, with a homodirectional waveform. The time to minimal systolic volume of the 16 left ventricular segments was consistent in the healthy group (Fig. 4). The time-volume curves of the 16 left ventricular segments appeared to be chaotic and irregular and presented with a heterodirectional waveform. The time to the minimal systolic volume point was different in patients implanted with DDD mode cardiac pacemakers (Fig. 5).

## Discussion

In the present study, we explored the feasibility of using RT3DE to assess the left ventricular systolic synchronicity, which proved to be practical and reproducible. By using RT3DE, it is possible to integrate the systolic function of 16 left ventricular segments into an index that may be used to assess the left ventricular dyssynchrony. Compared with the healthy group, indices were significantly different in patients implanted with DDD mode cardiac pacemakers.

Following the renewal and development of pacemakers, the importance of cardiac hemodynamics in patients implanted with pacemakers has been recognized. Cardiac pacemakers are able, not only to ameliorate the bradycardiac symptom, but also to supply an individual, optimal hemodynamics effect pacing system for patients. Therefore, the evaluation of cardiac function in patients implanted with cardiac pacemakers is the principal research subject of cardiac pacing.

The evaluation of left ventricular systolic synchrony (6–9) includes M-mode echocardiography (10), two-dimensional echocardiography (11), color Doppler echocardiography (12), Doppler tissue imaging (13) and tissue tracing imaging (14,15). However, the widespread application of these techniques has been limited by analysis that may lead to poor reproducibility. Although Doppler tissue imaging has a more accurate spatial resolution, it cannot provide data for the 16 left ventricular segments in a cardiac cycle.

RT3DE combines a quantitative borderline tracking technique (3) and the production of data of the left ventricular volume and cardiac function. Quantification of mechanical dyssynchrony with RT3DE takes all left ventricular segments into account by assessing the regularity of the time-volume variation in the left ventricular segments using indices based on three-dimensional regional volume changes. Zhang *et al* (16) demonstrated that when cardiac pacemakers were turned on and off, respectively, there were significant differences in the T-SD and Tmax of 16 and 12 segments, respectively, in patients with cardiac resynchronization. The standard deviation of the time to peak velocities of 12 segments achieved correlated closely with 16 segments Tmax (r, 0.809; P<0.05) and T-SD (r, 0.739; P<0.05). Using RT3DE, we found that the time-volume curves of the 16 segments appeared ordered and regular, with homodirectional waveforms. The time to minimal systolic volume point in left ventricular 16 segments was consistent in the healthy group. Findings of the present study indicated that the sequence that causes left ventricular myocardial contraction is associated with the regularity of the contraction. Normal myocardial contraction begins at the septum between the right and left ventricles, then moves through the cardiac apex, walls of the ventricles and ends at the base of the heart. Therefore, the time to minimal systolic volume point of left ventricular 16 segments was consistent.

Normal cardiac contraction depends on regular activation as well as the internal cardiac conductive system. Right ventricular pacing may cause the abnormality of electrodepolarization and mechanical contraction. Yu *et al* (17) observed that in patients with normal systolic function, conventional right ventricular apical pacing resulted in adverse left ventricular remodeling and in a reduction in the left ventricular ejection fraction. Prinzen *et al* (18) demonstrated that compared with left ventricular pacing, the left ventricular stroke volume is lower compared to pacing in the right ventricular apex. Johnson *et al* (19) showed that when ventricular pacing occurs, the region nearest to the pacing point was activated first, followed by the myocardium generating adverse motion. Moreover, compared to atrium pacing, ventricular pacing induced a dp/dt decrease of 27%. Thambo *et al* (20) demonstrated that the right ventricular apex was not the best pacing modality due to the discrepancy of the ventricular synchronicity.

In the present study, LESV was significantly increased (P<0.05), SV and LVEF were significantly decreased (P<0.05) and T-SD and Tmax were significantly prolonged (P<0.05) in patients implanted with DDD mode cardiac pacemakers. The time-volume curves of the 16 left ventricular segments presented a heterodirectional waveform and the time to the minimal systolic volume point differed in patients implanted with DDD mode cardiac pacemakers. The results indicated that right ventricular pacing may decrease left ventricular coordinated motion and systolic function.

Pacing from the right ventricular apex in DDD pacemakers involved first activating the right ventricular apex and propagating it within the interventricular septum. Subsequently, it was transferred to the free wall of the right and left ventricles, ending at the basilar part of the left ventricle. The sequence of ventricular activation and modality of motion was opposed to sinus rhythm, which produced an abnormal blood shunt in the chambers. Pacing at the right ventricular apex caused regional precontraction and myocardial strain of the right ventricular apex and adjacent regions. The blood in the ventricle flowed to the free wall and basilar part of the left ventricle, causing apical aneurysm, dilation, asynchronism and paradoxical motion. When activation propagated from the cardiac basilar region, the myocardial strain of the cardiac base and peripheral myocardium impulsed the blood to the apex, the left ventricular apex became swollen and bulged, similar to a ventricular aneurysm. When the interventricular septum became depolarized and contracted, the left ventricular free wall depolarized and then contracted. When the interventricular septum was at diastole, the strain originated from the contraction of the left ventricular free wall and the blood flow pressure in the ventricle reversed the direction and dilation of the interventricular septum. The incompatible systole and diastole of the regional ventricular myocardium, as well as the paradoxical motion caused abnormal blood shunt in the ventricle, while the global, prompt and synchronous left ventricular contraction changed into a torpid, incompatible segmental left ventricular contraction. Accordingly, systole was prolonged, the left ventricular ejection period was shortened, the stroke volume was reduced and the left ventricular ejection fraction decreased.

This study was designed to validate RT3DE as a method of quantifying myocardial systolic synchrony of the left ventricle in patients implanted with DDD mode cardiac pacemakers and healthy subjects. The RT3DE analysis and manual tracing endocardium outline of the left ventricle are subjective and depend on imaging quality. Thus, a totally blinded analysis would be preferable. The limitation of a full-time 60x60° sector may induce volume envelope insufficiency in patients with cardiac dilation and apex bulging. The accuracy of our method for quantifying left ventricular systolic synchrony was carried out by using RT3DE, but with no comparison with a gold standard. In the absence of a gold standard, the clinical value of each technique should be compared to establish the accuracy and clinical utility of each technique.

In conclusion, RT3DE is a feasible and reproducible method to quantify left ventricular function and systolic synchrony. Asynchronization of the left ventricular systolic performance was observed in patients with DDD mode cardiac pacemakers. The T-SD and Tmax of the 16 left ventricular segments measured by RT3DE may become effective indices in assessing ventricular systolic synchronism. The time-volume curves of the 16 left ventricular segments may reflect the regularity of ventricular systolic synchronism as well as show the time to minimal systolic volume point in all segments.

## Figures and Tables

**Table I:** Clinical and left ventricular function parameters.

Group	n	Age (years)	HR (bpm)	LEDV (ml)	LESV (ml)	SV (ml)	LVEF (%)
Healthy	20	62.8±5.5	67.7±6.2	73.13±16.81	25.27±7.71	47.87±11.81	65.22±6.31
DDD	20	66.6±7.2	66.4±5.8	82.72±30.96	42.95±23.79^a^	38.43±9.60^a^	48.08±9.79^a^

Values are the mean ± SD.

a DDD group vs. healthy group are significantly different (P<0.05); all other values without symbols are not significant. HR, heart rate (beats per minute); LEDV, left ventricular end-diastolic volume; LESV, left ventricular end-systolic volume; SV, stroke volume; LVEF, left ventricular ejection fraction; DDD, dual chamber.

**Table II:** Comparison of LV myocardial contractive synchronism in the two groups.

Group	Tmean (msec)	T-SD (msec)	Tmax (msec)
Healthy	421.90±47.30	10.00±3.62	36.60±14.90
DDD	443.45±43.05	29.00±7.31^a^	135.70±38.65^a^

Values are the mean ± SD.

a DDD group vs. healthy group are significantly different (P<0.05); all other values without symbols are not significant. Tmean, the mean value of the time to the point with minimal systolic volume of 16 left ventricular segments; T-SD, the standard deviation of Tmean; Tmax, the maximal difference of the time to the point of minimal systolic volume of 16 left ventricular segments; DDD, dual chamber.
